# Integrated CNV-seq, karyotyping and SNP-array analyses for effective prenatal diagnosis of chromosomal mosaicism

**DOI:** 10.1186/s12920-021-00899-x

**Published:** 2021-02-25

**Authors:** Na Ma, Hui Xi, Jing Chen, Ying Peng, Zhengjun Jia, Shuting Yang, Jiancheng Hu, Jialun Pang, Yanan Zhang, Rong Hu, Hua Wang, Jing Liu

**Affiliations:** 1 Department of Medical Genetics, Hunan Provincial Maternal and Child Health Care Hospital, Changsha, 410008 Hunan China; 2 National Health Commission Key Laboratory of Birth Defects Research, Prevention and Treatment, Changsha, 410008 Hunan China

**Keywords:** Chromosomal microarray analysis (CMA), Copy number variation sequencing (CNV‐seq), Copy number variations (CNVs), Prenatal diagnosis, Mosaicism

## Abstract

**Background:**

Emerging studies suggest that low‐coverage massively parallel copy number variation sequencing (CNV-seq) more sensitive than chromosomal microarray analysis (CMA) for detecting low-level mosaicism. However, a retrospective back-to-back comparison evaluating accuracy, efficacy, and incremental yield of CNV-seq compared with CMA is warranted.

**Methods:**

A total of 72 mosaicism cases identified by karyotyping or CMA were recruited to the study. There were 67 mosaic samples co-analysed by CMA and CNV-seq, comprising 40 with sex chromosome aneuploidy, 22 with autosomal aneuploidy and 5 with large cryptic genomic rearrangements.

**Results:**

Of the 67 positive mosaic cases, the levels of mosaicism defined by CNV-seq ranged from 6 to 92% compared to the ratio from 3 to 90% by karyotyping and 20% to 72% by CMA. CNV-seq not only identified all 43 chromosomal aneuploidies or large cryptic genomic rearrangements detected by CMA, but also provided a 34.88% (15/43) increased yield compared with CMA. The improved yield of mosaicism detection by CNV-seq was largely due to the ability to detect low level mosaicism below 20%.

**Conclusion:**

In the context of prenatal diagnosis, CNV-seq identified additional and clinically significant mosaicism with enhanced resolution and increased sensitivity. This study provides strong evidence for applying CNV-seq as an alternative to CMA for detection of aneuploidy and mosaic variants.

## Background

Chromosomal mosaicism is defined by the presence of two or more cell populations within the body and results from either gamete meiotic or mitotic cleavage-stage errors in the early preimplantation embryo [1]. Based on the differentiation stage when mosaicism arises, the aneuploid cells can reside only in extra‐fetal tissues (e.g. the placenta), only in the fetus, or in both. Therefore, mosaicism and the level detected has an important impact on the phenotype of first generation carriers but also on the recurrence risk with implications for prenatal counselling [2].

Karyotyping, with a maximum resolution of 3 Mb [3], has been used as the golden standard for identifying chromosomal abnormalities in prenatal diagnosis for more than 50 years. In general, the lower limit of true mosaicism detectable by karyotyping is around 5% [4]. However, in some cases, low level mosaicism can be due to culture artifacts. Chromosomal microarray (CMA) conducted on uncultured fetal cells from chorionic villus sampling or amniocentesis has gradually replaced conventional karyotyping for all prenatal diagnosis indications owing to a higher diagnostic yield, quicker turnaround time and elimination of cultural artifacts (pseudo mosaicism) [5]. Although it has been demonstrated to be a powerful tool to detect mosaicism at levels as low as 5% [6], it still remains difficult to detect mosaicism in clinical research when the ratio of euploid to aneuploid cells is below 20%. This is mainly due to platform differences, quality of the biopsy samples and maternal cell contamination (MMC). Besides, the efficiency to detect segmental mosaicism can be limited by probe design and genome location.

More recently, low‐coverage massively parallel copy number variation sequencing (CNV-seq) has emerged as a high-resolution and low-cost technology for detecting CNVs in clinical samples [5]. CNV‐seq can detect structural abnormalities larger than 100 kb and mosaicism as low as 5% [7, 8]. More and more studies have supported a higher sensitivity for low-pass GS in identifying low-level mosaicisms of both numerical disorders and submicroscopic rearrangements compared with routine CMA [5, 9, 10]. However, there is limited retrospective back-to-back comparison study to evaluate the accuracy and efficacy of CNV-seq compared with CMA has been reported in routine prenatal diagnosis. Herein, we conducted a study to evaluate the diagnostic outcome and technical limitations of CMA and CNV-seq for detection of mosaicism.

## Methods

### Study subjects

Prenatal diagnosis by karyotyping or CMA identified 72 fetuses with chromosome mosaicism from routine clinical samples collected in the Department of Medical Genetics of Hunan Provincial Maternal and Child Health Care Hospital between May 2018 to November 2019. The primary prenatal indications for the 72 women were: 21 (29%) for advanced maternal age (AMA, > 35 years), 13 (18%) with abnormal ultrasound structure scans (aUS), 19 (26%) with a high-risk maternal serum screening (hMSS) results, 54 (69%) high-risk z-scores for T21/T18/T13 by noninvasive prenatal screening (NIPS) and 3 (4%) had poor fertility histories (see Table 1 for case details).

**Table 1:** Chromosome, CMA and CNV-seq results on mosaic cases of autosomal aneuploidies, sex chromosome aneuploidy and large cryptic genomic rearrangements

Number	Case No	Sample type	Age	Indication	Chromosome (culture) Result	Copy number of reference chromosome		
						Karyotyping	CMA	CNV-seq
Mosaic cases of autosomal aneuploidies								
1	2	AF	28	Abnormal NIPS (T21)	47, XX, + 21[21]/46, XX [79]	2.21 (Chr 21)	2.29 (Chr 21)	2.22 (Chr 21)
2	4	AF	43	AMA, hMSS (T21), abnormal NIPS (T21)	47, XY, + 21[13]/46, XY [37]	2.26 (Chr 21)	2.20 (Chr 21)	2.20 (Chr 21)
3	6	AF	36	AMA, abnormal NIPS (T21)	47, XX, + 21[22]/46, XX [78]	2.22 (Chr 21)	2.29 (Chr 21)	2.27 (Chr 21)
4	7	AF	30	Abnormal NIPS (T21)	47, XX, + 21[45]/46, XX [5]	2.90 (Chr 21)	3.00 (Chr 21)	2.92 (Chr 21)
5	8	AF	31	Abnormal NIPS (T21)	47, XY, + 21[31]/47, XY [19]	2.62 (Chr 21)	2.72 (Chr 21)	2.79 (Chr 21)
6	12	AF	39	AMA, hMSS (T21)	47, XX, + 21[41]/46, XX [59]	2.82 (Chr 21)	3.00 (Chr 21)	2.78 (Chr 21)
7	13	AF	34	aUS (talipes valgus)	47, XY, + 18[33]/46, XY [17]	2.66 (Chr 18)	2.64 (Chr 18)	2.56 (Chr 18)
8	14	AF	39	AMA, abnormal NIPS (T18)	47, XY, + 18[44]/46, XY [6]	2.88 (Chr 18)	2.78 (Chr 18)	2.67 (Chr 18)
9	16	AF	41	AMA, abnormal NIPS (T15)	47, XX, + 15[4]/46, XX [96]	2.04 (Chr 15)	2.30 (Chr 15)	2.32 (Chr 15)
10	17	AF	39	AMA, abnormal NIPS (T15)	47, XY, + 15[12]/46, XY [88]	2.12 (Chr 15)	2.49 (Chr 15)	2.36 (Chr 15)
11	19	AF	36	AMA, abnormal NIPS (T22)	47, XX, + 22,1qh^+^[4]/46, XX, 1qh^+^[96]	2.04 (Chr 22)	2.22 (Chr 22)	2.16 (Chr 22)
12	22	AF	34	hMSS (T18), aUS (IUGR)	47, XX, + 2[3]/46, XX [97]	2.03 (Chr 2)	2.28 (Chr 2)	2.22 (Chr 2)
13	1	AF	27	Abnormal NIPS (T21)	47, XY, + 21[4]/46, XY [96]	2.04 (Chr 21)	Normal	2.10 (Chr 21)
14	3	AF	33	Abnormal NIPS (T21)	47, XY, + 21[6]/46, XY [94]	2.06 (Chr 21)	Normal	2.14 (Chr 21)
15	5	AF	32	Abnormal NIPS (T21)	47, XX, + 21[9]/46, XX [91]	2.09 (Chr 21)	Normal	2.07 (Chr 21)
16	9	AF	31.5	Abnormal NIPS(T10)	47, XX, + 21[3]/46, XX [97]	2.03 (Chr 21)	Normal	2.08 (Chr 21)
17	10	AF	24	hMSS (T21)	47, XX, + 21[3]/46, XX [97]	2.03 (Chr 21)	Normal	2.05 (Chr 21)
18	15	AF	41	AMA, abnormal NIPS (T13)	47, XY, + 13[16]/46, XY [84]	2.16 (Chr 13)	Normal	2.10 (Chr 13)
19	11	AF	39	AMA, abnormal NIPS (T21)	47, XY, + 21[5]/46, XY [95]	2.05 (Chr 21)	Normal	Normal
20	20	AF	28	aUS (increased NF)	47, XY, + 9[7]/46, XY [43]	2.14 (Chr 9)	Normal	Normal
21	21	AF	31	Previous pregnancy with CHD	47, XX, + 20[12]/46, XX [58]	2.17 (Chr 20)	Normal	Normal
22	18	AF	31	Abnormal NIPS (T8)	46, XX, 9qh +	Normal(Chr 8)	2.24 (Chr 8)	2.18 (Chr 8)
Mosaic cases of sex chromosome aneuploidy								
23	24	AF	31	Abnormal NIPS (X-)	45, X [2]/46, XX [48]	1.96(Chr X)/0 (Chr Y)	1.80 (Chr X)/0 (Chr Y)	1.87 (Chr X)/0 (Chr Y)
24	29	CB	33	aUS (oligohydramnios)	45, X [8]/46, XX [95]	1.92 (Chr X)/0 (Chr Y)	1.80 (Chr X)/0 (Chr Y)	1.82 (Chr X)/0 (Chr Y)
25	31	AF	28	Abnormal NIPS (X-)	45, X [13]/46, XX [87]	1.87 (Chr X)/0 (Chr Y)	1.80 (Chr X)/0 (Chr Y)	1.85 (Chr X)/0 (Chr Y)
26	33	AF	30	Abnormal NIPS (X-)	45, X [15]/46, XX [85]	1.85(Chr X)/0 (Chr Y)	1.78 (Chr X)/0 (Chr Y)	1.82 (Chr X)/0 (Chr Y)
27	34	AF	31	Abnormal NIPS (X-)	45, X [8]/46, XX [42]	1.84(Chr X)/0 (Chr Y)	1.62 (Chr X)/0 (Chr Y)	1.68 (Chr X)/0 (Chr Y)
28	35	AF	30	hMSS (T21), abnormal NIPS (X-)	45, X [16]/46, XY [84]	1.84(Chr X)/0 (Chr Y)	1.80 (Chr X)/0 (Chr Y)	1.79 (Chr X)/0 (Chr Y)
29	38	AF	38	AMA, abnormal NIPS (X-)	45, X [15]/46, XX [65]	1.81(Chr X)/0 (Chr Y)	1.43 (Chr X)/0 (Chr Y)	1.48 (Chr X)/0 (Chr Y)
30	39	AF	28	hMSS (T21), abnormal NIPS (X-)	45, X [20]/46, XX [80]	1.80(Chr X)/0 (Chr Y)	1.74 (Chr X)/0 (Chr Y)	1.72 (Chr X)/0 (Chr Y)
31	40	AF	28	Abnormal NIPS (X-)	45, X [23]/46, XX [77]	1.77(Chr X)/0 (Chr Y)	1.62 (Chr X)/0 (Chr Y)	1.54 (Chr X)/0 (Chr Y)
32	42	AF	29	Abnormal NIPS (X-)	45, X [29]/46, XX [71]	1.71(Chr X)/0 (Chr Y)	1.73 (Chr X)/0 (Chr Y)	1.79 (Chr X)/0 (Chr Y)
33	44	AF	42	AMA, hMSS (T21), abnormal NIPS (X-)	45, X [16]/46, XX [84]	1.84(Chr X)/0 (Chr Y)	1.80 (Chr X)/0 (Chr Y)	1.84 (Chr X)/0 (Chr Y)
34	45	AF	28	hMSS (T21), abnormal NIPS (X-)	45, X [13]/46, XX [27]	1.68(Chr X)/0 (Chr Y)	1.45 (Chr X)/0 (Chr Y)	1.53 (Chr X)/0 (Chr Y)
35	47	AF	37	AMA, aUS(increased NT and NF)	45, X [51]/46, XY [9]	1.00(Chr X)/0.15 (Chr Y)	1.00 (Chr X)/0.5 (Chr Y)	1 (Chr X)/0.45 (Chr Y)
36	48	AF	32	hMSS (T21), abnormal NIPS (X-)	45, X [27]/47, XXX [23]	1.92 (Chr X)/0 (Chr Y)	1.70 (Chr X)/0 (Chr Y)	1.83 (Chr X)/0 (Chr Y)
37	49	AF	28	Abnormal NIPS (X-)	45, X [34]/47, XXX [16]	1.64 (Chr X)/0 (Chr Y)	1.53 (Chr X)/0 (Chr Y)	1.70 (Chr X)/0 (Chr Y)
38	50	AF	32	Abnormal NIPS (X-)	45, X [48]/47, XXX [2]	1.08 (Chr X)/0 (Chr Y)	1.20 (Chr X)/0 (Chr Y)	1.46 (Chr X)/0 (Chr Y)
39	51	AF	28	aUS(increased NT)	45, X [32]/47, XXX [18]	1.72 (Chr X)/0 (Chr Y)	1.40 (Chr X)/0 (Chr Y)	1.59 (Chr X)/0 (Chr Y)
40	52	AF	33	hMSS (T21), abnormal NIPS (X-)	45, X [23]/47, XXX [77]	2.54 (Chr X)/0 (Chr Y)	2.51 (Chr X)/0 (Chr Y)	2.58 (Chr X)/0 (Chr Y)
41	53	AF	40	AMA, previous pregnancy with DMD	45, X [12]/47, XYY [88]	1.00 (Chr X)/1.76 (Chr Y)	1.00 (Chr X)/2.00 (Chr Y)	1 (Chr X)/1.59 (Chr Y)
42	55	AF	28	hMSS (T21), abnormal NIPS (X-)	45, X [17]/47, XXX [10]/ 46, XX [23]	1.86(Chr X)/0 (Chr Y)	2.22 (Chr X)/0 (Chr Y)	2.18 (Chr X)/0 (Chr Y)
43	57	AF	37	AMA, abnormal NIPS (X +)	47, XXY [31]/46, XY [19]	1.62 (Chr X)/1.00 (Chr Y)	1.58 (Chr X)/1.00 (Chr Y)	1.63 (Chr X)/1.00 (Chr Y)
44	58	AF	25	Abnormal NIPS (T9)	47, XXY [39]/46, XY [11]	1.78 (Chr X)/1.00 (Chr Y)	1.53 (Chr X)/1.00 (Chr Y)	1.66 (Chr X)/1.00(Chr Y)
45	59	AF	38	AMA, abnormal NIPS (X +)	47, XXY [46]/46, XY [4]	1.92 (Chr X)/1.00 (Chr Y)	2 (Chr X)/1.00 (Chr Y)	2 (Chr X)/1.00 (Chr Y)
46	60	AF	30	Abnormal NIPS (X +)	47, XXY [30]/46, XY [20]	1.60 (Chr X)/1.00 (Chr Y)	1.64 (Chr X)/1.2 (Chr Y)	1.70 (Chr X)/1.00 (Chr Y)
47	62	AF	29	hMSS (T21)	47, XYY [26]/46, XY [24]	1.00 (Chr X)/1.52 (Chr Y)	1(Chr X)/1.70 (Chr Y)	1(Chr X)/1.76 (Chr Y)
48	25	AF	31	Abnormal NIPS (X-)	45, X [4]/46, XX [96]	1.96(Chr X)/0 (Chr Y)	Normal	1.89 (Chr X)/0 (Chr Y)
49	28	AF	34	hMSS (T21), abnormal NIPS (X-)	45, X [7]/46, XX [93]	1.93 (Chr X)/0 (Chr Y)	Normal	1.92 (Chr X)/0 (Chr Y)
50	36	AF	39	AMA, hMSS (T21), abnormal NIPS (X-)	45, X [13]/46, XX [67]	1.84(Chr X)/0 (Chr Y)	Normal	1.92 (Chr X)/0 (Chr Y)
51	37	CB	30	Abnormal NIPS (X-), aUS(IUGR)	45, X [19]/46, XY [86]	1.00(Chr X)/0.82 (Chr Y)	Normal	1.00(Chr X)/0.92 (Chr Y)
52	41	AF	27	hMSS (T21), abnormal NIPS (X-)	45, X, 1qh^+^ [24]/ 46, XX, 1qh^+^ [76]	1.76(Chr X)/0 (Chr Y)	Normal	1.92 (Chr X)/0 (Chr Y)
53	43	AF	39	AMA, failed NIPS	45, X [19]/46, XY [42]	1.00(Chr X)/0.69 (Chr Y)	Normal	1 (Chr X)/0.77 (Chr Y)
54	46	AF	28	Abnormal NIPS (X-)	45, X [6]/46, XX [94]	1.94(Chr X)/0 (Chr Y)	Normal	1.92 (Chr X)/0 (Chr Y)
55	54	CB	22	aUS (oligohydramnios and abnormal soft marker)	45, X [29]/47, XYY [51]	1.00 (Chr X)/1.28 (Chr Y)	Normal	1 (Chr X)/0.8 (Chr Y)
56	56	AF	35	AMA, abnormal NIPS (X +)	47, XXY [28]/46, XY [72]	1.28 (Chr X)/1.00 (Chr Y)	Normal	1.15 (Chr X)/1.00 (Chr Y)
57	23	AF	32	aUS (increased NF), Abnormal NIPS (X-)	45, X [4]/46, XX [100]	1.96(Chr X)/0 (Chr Y)	Normal	Normal
58	26	AF	23	hMSS (T18), abnormal NIPS (X-)	45, X [5]/46, XX [95]	1.95 (Chr X)/0 (Chr Y)	Normal	Normal
59	27	AF	38	AMA	45, X [1]/46, XY [49] 45,X[4]/46,XY[69] ^**ǂ**^	1.98 (Chr X)/0 (Chr Y)	Normal	Normal
60	30	AF	37	AMA, abnormal NIPS (X-)	45, X [9]/46, XX [91]	1.91 (Chr X)/0 (Chr Y)	Normal	Normal
61	32	AF	28	Previous pregnancy with duplication of 11p15.5p15.4 (3.6 Mb)	45, X [14]/46, XY [90]	1.00 (Chr X)/0.87 (Chr Y)	Normal	Normal
62	61	CB	31	Abnormal NIPS (X +)	47, XXX [3]/46, XX [47]	2.06 (Chr X)/0 (Chr Y)	Normal	Normal
Mosaic cases of large cryptic genomic rearrangements								
63	63	CB	33	hMSS (T21), aUS (VSD)	46, XX	/	arr[hg19]8p21.3p11.21(19,832,920–41,268,763) × 2.23	seq[hg19] dup(8)(p22-p11.1) chrX:g.18500000–43,800,000 dup 25.3 Mb × 2.23
64	64	AF	29	hMSS (T21), abnormal NIPS (X-)	45, X [25]/46, X, del(X)(q21.2) [25]	/	arr[hg19] Xq21.31q28(91,397,723–155,233,098) × 1 arr [hg19] Xp2.33q21.31(1,832,912–87,597,750) × 1.51 arr[hg19](Y) × 0	seq[hg19] dup(X)(p22.33q21.31) chrX:g.2700000–90,720,000 dup 84.86 Mb × 1.53 seq[hg19] dup(X)(q21.31q28) chrX:g.90720000–154,940,000 dup 64.22 Mb × 1.042 seq[hg19](Y) × 0
65	65	AF	23	aUS (increased NT)	46, X, Yp^+^ [29]/45, X [21]	/	arr[hg19]Yq11.222q11.223(20,885,550–24,889,194) × 2	seq[hg19] dup(Y)(q11.221q11.223) chrY:g.19,520,000–24,520,000 dup 5.00 Mb × 2
66	66	AF	29	Abnormal NIPS (X-)	46, X, + mar [34]/45, X [16]	/	arr[hg19] Xp22.33p11.21 (168,551–55,476,636) × 1 arr[hg19] Xq13.2q28 (72,016,380–155,233,098) × 1 arr[hg19](Y) × 0	seq[hg19] del(X)(p22.33p11.21) chrX:g.2,700,000–55,560,000 del 52.86 Mb × 1.042 seq[hg19] del(X)(q13.2q28) chrX:g.72,200,000–154,940,000 del 82.74 Mb × 1.05 arr[hg19](Y) × 0
67	67	AF	27	Abnormal U/S (increased NT)	48, XX, + idic(X)(p11.2), + 21 [43]/47, XX, + 21 [7]	/	arr(21) × 3 arr[hg19] Xp11.21q28 (57,435,464–155,233,098) × 4	seq[hg19]dup(21)(q11.2q22.3) chr21:g.14300000_48129895 dup 32.26 Mb × 2.971 seq[hg19]dup(X)(p22.31q28) chrX:g.6100000–154,940,000 dup 93.72 Mb × 3.111

CMA, chromosomal microarray analysis; AF, amniotic fluid; AMA, advance maternal age; aUS, abnormal ultrasound; NT, nuchal translucency; T21, trisomy 21; hMSS, high-risk of maternal serum screening; NIPS, non-invasive prenatal screening; T13, trisomy 13; CHD, congenital heart disease; IUGR, intrauterine growth retardation; N/A, not available

ǂKaryotypes from two independent laboratories. (pseudo mosaicism)

### Sample preparation

Genomic DNA (gDNA) was extracted from amniotic fluid (approximately 8 mL) or fetal cord blood (approximately 200µL) by using DNA Extraction Kit (Tissue and cells) and QIAamp DNA Blood Mini Kit (QIAGEN, Hilden, Germany) respectively. The quality and concentration of gDNA from the samples was assessed using the Qubit 2.0 Fluorometer (Thermo Fisher Scientific, Waltham, Massachusetts, USA). Multiplex fluorescent PCR using 21 short tandem repeat (STR) markers was performed using the MicroreaderTM 21 Direct ID System (Suzhou Yuewei Gene Technology corporation, China) to measure MMC and identify polyploidy [11]. All 72 samples had MCC levels less than 5% and qualified for CMA and CNV-seq analysis.

### Karyotyping

Amniotic fluid and fetal cord blood samples were obtained under sterile conditions. For all prenatal samples, two cell cultures were set up by trypsin-Giemsa banding, seeding the flasks with either 10 mL of amniotic fluid or 0.2 mL of cord blood. Amniotic and cord blood cultures were expanded for 8 and 3 days, respectively, and then G-banded (320–400 bands) karyotyping analyses were performed on metaphases cells according to standard protocols. According to established guidelines [12], analysis of at least 50 metaphases cells was used to diagnose mosaicism. Mosaicism was defined as either level I/II pseudomosaicism or level III true mosaicism. Level I pseudomosaicism denotes the presence of a single aneuploid cell whereas level II pseudomosaicism denotes two or more aneuploid cells from one primary culture only. Level III is the presence of multiple aneuploid cells from at least two primary cultures and was classified as true mosaicism. In this study, with the exception of case 27, all mosaic samples were diagnosed with level II true mosaicism.

### CMA analysis

SNP array analysis was performed using Affymetrix CytoScan®750 K Array (Affymetrix Inc, CA, USA), according to the manufacturers protocol. Array results were analyzed using Chromosome Analysis Suite Software (ChAS; version 4.0). All genomic coordinates were taken from the February 2009 (hg19) human reference sequence (NCBI Build 37). Genes and Online Mendelian Inheritance in Man (OMIM) references were from RefSeq and OMIM entries, respectively. The theoretical values for the detection of a single copy gain or loss were applied as previously reported [13, 14].

### CNV‐seq analysis

Genomic DNA (10 ng) was fragmented and DNA library was constructed as previously described [7]. Multiple libraries were indexed and pooled into a single lane and sequenced on the Nextseq CN500 instrument (Illumina, Inc.) to produce approximately 5 million single-end reads of 45 bp (including the 8 bp index sequence). For each sample, approximately 2.8–3.2 million uniquely and precisely mapped 36 bp reads were aligned to the human reference genome using the Burrows–Wheeler mapping algorithm [15] and then allocated to 20-kb bins sequentially across each of 24 chromosomes. Binned read data of all samples were compared internally with each other as described previously [8], and then log2 of the mean CNV of each sequencing bin along the length of each chromosome was plotted with log2[0] representing two copies (normal), log2[1.5] three copies (duplication) and log2[0.5] one copy (deletion). Trisomic mosaicism was defined by a mean chromosome copy number between 2.05 (5%) and 2.95 (95%) whereas monosomic mosaicism was defined as a mean chromosome copy number between 1.05 (5%) and 1.95 (95%).

## Results

**Fig. 1:**
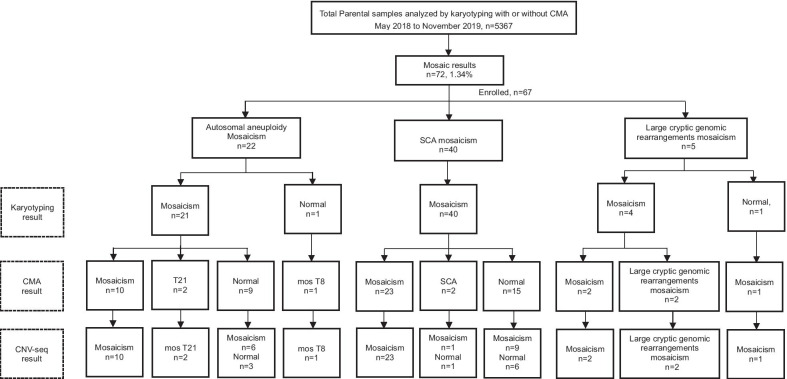
Flowchart of mosaicism analysis by copy number variations sequencing (CNV-seq) versus chromosomal microarray (CMA). Karyotyping was performed in 5,367 prenatal cases

Following prenatal diagnosis of 5,367 pregnancies with karyotyping and CMA, 72 fetuses were identified with mosaic results, including 22 with autosomal aneuploidy (30%), 40 with sex chromosome aneuploidy (n = 56%) and 10 with large cryptic genomic rearrangements (14%). Five samples with large cryptic genomic rearrangements were excluded from the analysis due to a lack of DNA following CMA testing (Additional file 1: Table S1). Therefore, 67 samples (40 sex chromosome aneuploidies, 22 autosomal aneuploidies and 5 large cryptic genomic rearrangements) were eventually analyzed by both CMA and CNV-seq (Fig. 1).

### Diagnostic concordance of CNV-seq and CMA versus karyotyping

For the 65 of 67 samples identified as mosaic by karyotyping, 41(63%) were also confirmed by CMA (Fig. 1). Of the 41 positives, 37 showed low levels of mosaicism around 20%. Two samples normal by karyotyping, we revealed as mosaic trisomy 8 and mosaic partial trisomy 8 by CMA. In comparison, CNV-Seq not only identified all 43 mosaics detected by CMA, but also identified an additional 15 mosaic samples, increasing the yield of mosaic detection by 35% over CMA. The levels of mosaicism defined by CNV-seq ranged from 6 to 92%. Further, the chromosomal map intervals, size, and copy number of the reportable mosaicisms detected by both DNA-based techniques were almost identical. Nine samples diagnosed as normal by CMA were also confirmed by CNV-seq.

### Chromosomal mosaicism for autosomal aneuploidy

For mosaic autosomal aneuploidy, there were 21 cases identified by karyotyping and 1 case by CMA. The majority of mosaic cases (16 of 22) were from high-risk noninvasive prenatal screening group (Table 1). The mosaics identified involved trisomy 21 (12, 55%), trisomy 18 (2, 9%), trisomy 15 (2, 9%) and other autosomal trisomies (6, 27%).

**Fig. 2:**
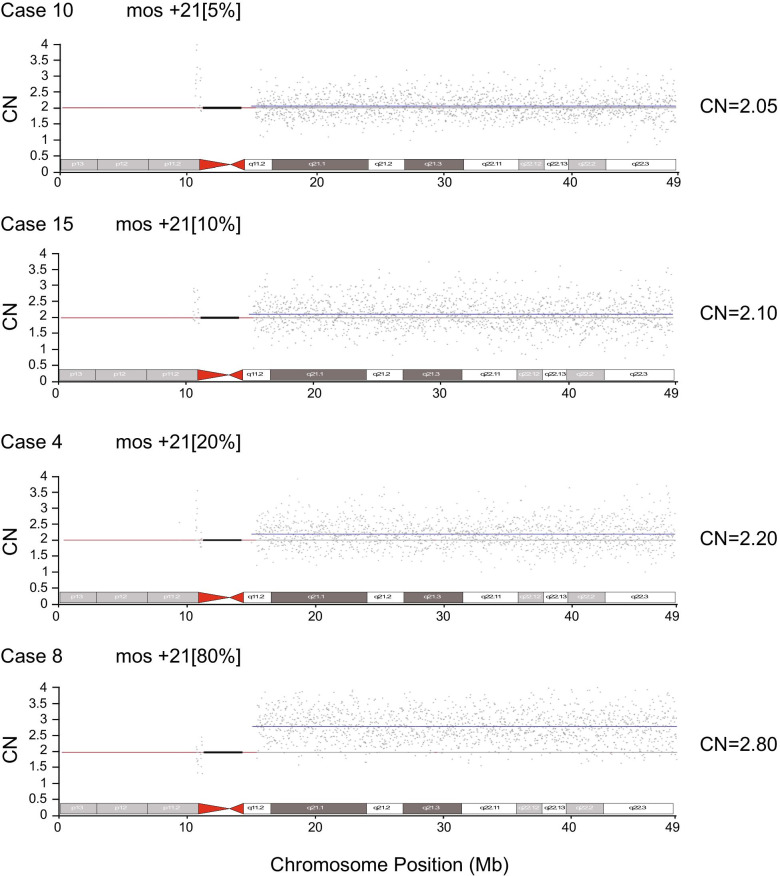
Examples of CNV-seq profiles for different levels of trisomy 21 mosaicism. CNV-Seq profiles are shown for Case 10, Case15, Case 4 and Case 8 with 5%, 10%, 20% and 79% trisomy 21 mosaicism, respectively. The blue line represents the mean copy number and the black box represents the centromere

CMA analysis identified 13 of 22 cases with mosaicism levels as low as 20% whereas CNV-seq identified 19 of the 22 cases with mosaicism at levels down to 5% (Fig. 2). For all 19 cases confirmed by CNV-seq, the percentages of trisomic cells for trisomy 21, 18 and 13 were in good close agreement with karyotyping results. However, for case 16, 17, 18, 19 and 22, the proportion of aneuploidy was much lower in culture samples compared with uncultured. Notably, for case 18, the mosaic trisomy 8 was not detected in the cultured AF sample by metaphase analysis of 100 G-banded cells whereas CMA and CNV-seq showed 24% and 18% trisomy 8 mosaicism, respectively (Additional file 1: Fig. S1). For the remaining three cases 11, 20 and 21, both CNV-seq and CMA showed a normal result in uncultured amniotic fluid cells but karyotype showed a mosaic pattern of trisomy 21, trisomy 9 and trisomy 20 in cultured amniotic fluid cells, respectively.

### Chromosomal mosaicism for sex chromosome aneuploidy

There were 40 cases with mosaic sex chromosome aneuploidies accounting for 60% of all cases identified by karyotyping. Clinical indications for these cases were broader including aUS (n = 6), AMA (n = 11), hMSS (n = 12), NIPS (n = 33) and poor fertility history (n = 2). Details of the sex chromosomes involved and the clinical course of the 40 pregnancies are presented in Table 1. The mosaic findings including 25 cases for monosomy X (45, X/46, XX), 7 cases for monosomy X and trisomy X (45, X /47, XXX) or monosomy X and disomy X (45, X /47, XXY), 5 cases for disomy X (47, XXY /46, XY), 1 case for disomy Y (47, XYY /46, XY), 1 case for trisomy X (47, XXX /46, XX) and 1 case for both monosomy X, disomy X, and trisomy X (45, X /46, XX/47, XXX).

Based on the karyotyping data, the percentage of monosomic or trisomic cells varied from as low as 4% to as high as 92% (Table 1). Of the 40 sex chromosome mosaics identified by karyotyping, CMA identified 23 cases of mosaicism (levels as low as 20%), 2 cases of whole sex chromosome aneuploidy and 15 normals. In contrast, CNV-seq identified 34 cases of mosaicism (level as low as 8%) with an incremental yield of mosaicism of 22.5% over CMA. There was a 100% positive concordance between CMA and CNV-Seq for 23 mosaic samples. It should be noted that in case samples 38, 47, 50, 54 and 55, the proportion of monosomy X or disomy Y varied by more than 30% in cultured samples compared with uncultured samples.

For the 9 additional cases of mosaicism identified by CNV-seq, the measured levels of mosaicism were low, ranging from 8 to 23%. Further, for cases 36 and 41, there were 1.92 and 1.90 haploid equivalents of chromosome X in the amniotic fluid samples, respectively, whereas CNV-seq analysis of available fetal placenta confirmed placental mosaicism with chromosome X of 1.17–1.87 and 1.3–1.85 haploid equivalents (Additional file 3: Fig.S2).

For cases 23, 26, 27, 30, 32 and 61, both CNV-seq and CMA showed a normal result in uncultured amniotic fluid cells, but karyotyping showed a mosaic pattern of monosomy X or disomy X in cultured amniotic fluid cells. Among these cases, karyotyping detected a mosaic pattern of monosomy X or disomy X of less than 10% in 5 of the 6 cases. The negative results of CMA and CNV-seq may have been due to technical limitations or culture artifacts by karyotyping.

### Chromosomal mosaicism for large cryptic genomic rearrangements

A total of 5 cases with large cryptic genomic rearrangements were identified by karyotyping. Of these, 4 (cases 64, 65, 66, 67) had a mosaic pattern involving a small supernumerary marker chromosome (sSMC) or unclarified derived chromosome. By CMA, the character, origin and pathogenicity of these sSMC was further clarified (Additional file 4: Fig. S3–Additional file 8: Fig. S7). Details of the chromosomes involved and the clinical course of the 5 pregnancies are presented in Table 1.

## Discussion

Extensive prenatal studies have shown that mosaicism can involve most of the chromosomes, presenting as trisomy, monosomy, triploidy, deletion, duplication and ring mosaics. A clinical cytogenetics laboratory performing prenatal diagnosis should therefore understand the limitations of cell-based chromosome analyses and DNA-based CNV-seq and CMA analyses for detecting and measuring the levels of mosaic aneuploidies and other cryptic genomic rearrangements.

To our knowledge, this is the first retrospective back-to-back study evaluating the efficacy of CNV-seq in detecting mosaicism, benchmarking against CMA and karyotyping as a reference. In our prenatal study, 72 of 5,367 cases showed a mosaic chromosome pattern with 1.39% (67/4825) detection rates among amniotic fluid samples and 0.92% (5/542) detection rates among cord blood samples. This rate is similar to the 1–2% chromosomal mosaicism rate in CVS [16, 17] but higher than the 0.1–0.5% rate in amniotic fluid samples [18, 19]. Among the 72 mosaic pattern fetuses, high-risk NIPS results (53, 68.91%) was the most common prenatal diagnosis indication. In NIPS analyses, there are occasional samples in gray zone for positive Z scores, indicating possible mosaicism. Thus, amniocentesis and karyotyping should be used to follow up these results to confirm full aneuploidy or mosaic aneuploidy [20]. If mosaicism is present, a more accurate assessment for levels can be obtained by CNV-seq.

The current study demonstrated that CNV-seq is more sensitive than CMA for identifying mosaicism, with the ability to detect levels down to 5%. This study confirms previous modelling of mosaicism where XXX and XO mosaicism was readily detectable at 5% [8]. Although SNP arrays has been demonstrated to be a powerful tool to detect mosaicism at levels as low as 5% by using IlluminaQuad610 array[6], the detectable levels are still variable among different CMA platform (9%-20% for array CGH [18, 21] and 30%-70% for Affymetrix arrays [22, 23]). Further, when using poor-quality, contaminated or fragmented DNA as the starting template, CNV-seq preforms much better than array CGH platforms for detection of aneuploidy and mosaicism [24]. In addition, for cryptic segmental mosaicism by CMA, the detection rate is not only due to size of the CNV but also influenced by nonuniform distribution of the probes in some genomic regions [25]. As an example, Wang et al.[5] previously showed a variable probe density in the targeted region among different CMA platforms, prevented detection of pathogenic 298.7-kb deletion in the *FBN2* gene that was detectable by low-pass genome sequencing. This reinforces the advantages of applying low-pass genome sequencing for CNV analysis which relies on genome-wide uniformly distributed reads mapped to sequential bins across all chromosomes.

Variable proliferation of cells with different karyotype under in vitro cell culture may have contributed to the inconsistent results between CNV-seq/CMA (uncultured samples) and karyotyping (cultured samples). Cell culture tends to promote the in vivo selection of euploid over aneuploid cells, which has been reported to increase with age of the culture [26]. In our study, the percentages of cells for trisomy 21, 18 and 13 by cell-based chromosome analyses were in good agreement with mosaicism levels measured by DNA-based CNV-seq or CMA and were compared. However, for mosaic trisomy 15, trisomy, trisomy 2 and trisomy 22, levels of mosaicism by CNV-Seq and CMA were higher than those seen by karyotyping, which is consistent with previous reports for autosomal mosaicism [27–30]. This supports the general notion that normal cells may have had a growth advantage in culture or the abnormal cell line may have a culture disadvantage [18]. The exception was monosomy X (7 of 30 cases), where the monosomy X cell line appeared to have a growth advantage over the normal cells, since CNV-seq and CMA measured monosomy X mosaicism at much lower levels. There were also 9 discordant cases where karyotyping detected mosaicism above 10%, but CNV-seq/CMA showed a normal result. Based on postnatal outcomes, a normal karyotype was confirmed, suggesting the mosaicism observed by karyotyping was due to low level culture artifacts. On balance, our studies highlight the advantage of using direct uncultured samples which can avoid artifact of culture, provides a quicker result and levels of mosaicism are more accurate to make a firm diagnosis.

## Conclusions

This study evaluated the effectiveness of CNV-Seq for detecting low-level mosaicism in prenatal diagnosis. The retrospective analysis found that CNV-seq identified additional and clinically significant information with enhanced resolution and increased sensitivity for mosaicism (35% increased yield) compared with CMA. The diagnosis and genetic counselling for mosaicism in a prenatal setting remains challenging. Based on our findings, we propose that low level mosaic findings from karyotyping should be confirmed with a DNA based method, preferably CNV-seq if available.

## Supplementary Information

**Additional file 1**.** Table S1**: Chromosome and CMA results on excluded mosaic cases of large cryptic genomic rearrangements..

**Additional file 2**.** Figure S1**: Case 18. Panel A. CMA analysis of uncultured AF samples shows mosaic trisomy 8 (~24%). Panel B. CNV‐seq profile of the same sample shows a slightly lower ratio of mosaic trisomy 8 (~18%). The blue line represents the mean copy number and the black box represents the centromere.

**Additional file 3**.** Figure S2**: Cases 36 and 41. Panel A. The CMA analysis of uncultured AF samples shows a normal result. The CNV‐seq profile of the same AF sample shows a low ratio of mosaic monosomy X (~8%). The CNV‐seq profiles of the maternal and fetal center of placenta show monosomy X with the level of 11% and 83% mosaicism. The blue line represents the mean copy number and the black box represents the centromere. Panel B. The CMA analysis on uncultured AF samples shows a normal result. The CNV‐seq profiles of the same AF samples shows a low ratio of mosaic monosomy X (~8%). The CNV‐seq profiles of the maternal and fetal center of placenta show monosomy X with the level of 15% and 78% mosaicism. The blue line represents the mean copy number and the black box represents the centromere.

**Additional file 4**.** Figure S3**. Case 63 (normal karyotype). CMA and CNV-seq results were discordant with karyotyping. Panel A. CMA analysis on uncultured cord blood sample shows a 20.44 Mb mosaic duplication at chromosome 8p21.3p11.21 (~ 23% of cells) marked by arrow. Panel B. CNV-seq shows a mosaic 8p22p11.1 duplication of 25.3 Mb (~23% of cells). Positions of CNVs are indicated by the dashed boxes.

**Additional file 5**.** Figure S4**: Case 64. Chromosomal mosaicism for Xp2.33q21.31 detected by CMA and CNV-Seq. Panel A. CMA result. Panel B. CNV-Seq result. Blue lines on sequencing plots represent mean copy number changes. Panel C. Karyotype showing Xp deletion.

**Additional file 6**.** Figure S5**: Case 65. Mosaic duplication of Yq11.222q11.223 detected by CMA and CNV-Seq. Panel A. CMA result. Panel B. CNV-Seq result. Blue lines on sequencing plots represent mean copy number changes. Panel C. Karyotype showing Yq duplication. Positions of CNVs are indicated by the dashed boxes.

**Additional file 7**.** Figure S6**: Case 66. Mosaic deletion of Xp22.33p11.21 and Xq13.2q28 detected by CMA and CNV-Seq. Panel A. CMA result. Panel B. CNV-Seq result. Blue lines on sequencing plots represent mean copy number changes. Panel C. Karyotype showing X deletion. Positions of CNVs are indicated by the dashed boxes.

**Additional file 8**. ** Figure S7**: Case 67. Mosaic trisomy 21 and mosaic duplication of Xp11.21q28 detected CMA and CNV-Seq. Panel A. CMA result. Panel B. CNV-Seq result. Blue lines on sequencing plots represent mean copy number changes. Panel C. Karyotype showing trisomy 21 and XXX. Positions of CNVs are indicated by the dashed boxes.

## Data Availability

NCBI human reference genome GRCh37 was used as the reference genome during the current study, which is available in National Center for Biotechnology Information (NCBI) at https://www.ncbi.nlm.nih.gov/projects/genome/guide/human/index.shtml. To ensure patient confidentiality, data containing potentially identifiable information was not shared. Raw data of CMA and CNV-seq assay obtained in our study is available from the corresponding author on reasonable request. All data generated or analysed during this study, without identifiable information, is available in this published article and its Supplemental Data Tables and Figures.

## Abbreviations

CNV-seq: Copy Number Variation Sequencing
CMA: Chromosomal Microarray Analysis
CNV: Copy Number Variation
AMA: Advanced Maternal Age
aUS: Abnormal Ultrasound Structure
hMSS: High-risk of Maternal Serum Screening
NIPS: Noninvasive Prenatal Screening
sSMC: Supernumerary Marker Chromosome
CPM: Confined Placental Mosaicism

## References

[1] Kuliev A, Verlinsky Y (2004). Meiotic and mitotic nondisjunction: lessons from preimplantation genetic diagnosis. Hum Reprod Update.

[2] Castera L, Gauthier-Villars M, Dehainault C, Michaux D, Benachi A (2011). Mosaicism in clinical practice exemplified by prenatal diagnosis in retinoblastoma. Prenat Diagn.

[3] Vermeesch JR, Fiegler H, de Leeuw N, Szuhai K, Schoumans J, Ciccone R (2007). Guidelines for molecular karyotyping in constitutional genetic diagnosis. Eur J Hum Genet.

[4] Hook EB (1977). Exclusion of chromosomal mosaicism: tables of 90%, 95% and 99% confidence limits and comments on use. Am J Hum Genet.

[5] Wang H, Dong Z, Zhang R, Chau MHK, Yang Z, Tsang KYC (2020). Low-pass genome sequencing versus chromosomal microarray analysis: implementation in prenatal diagnosis. Genet Med.

[6] Conlin LK, Thiel BD, Bonnemann CG, Medne L, Ernst LM, Zackai EH (2010). Mechanisms of mosaicism, chimerism and uniparental disomy identified by single nucleotide polymorphism array analysis. Hum Mol Genet.

[7] Liang D, Peng Y, Lv W, Deng L, Zhang Y, Li H (2014). Copy number variation sequencing for comprehensive diagnosis of chromosome disease syndromes. J Mol Diagn.

[8] Wang Y, Chen Y, Tian F, Zhang J, Song Z, Wu Y (2014). Maternal mosaicism is a significant contributor to discordant sex chromosomal aneuploidies associated with noninvasive prenatal testing. Clin Chem.

[9] Chaubey A, Shenoy S, Mathur A, Ma Z, Valencia CA, Reddy NB (2020). Low-pass genome sequencing: validation and diagnostic utility from 409 clinical cases of low-pass genome sequencing for the detection of copy number variants to replace constitutional microarray. J Mol Diagn.

[10] Chau M, Wang H, Lai Y, Zhang Y, Xu F, Tang Y (2020). Low-pass genome sequencing: a validated method in clinical cytogenetics. Hum Genet.

[11] Liu S, Song L, Cram DS, Xiong L, Wang K, Wu R (2015). Traditional karyotyping vs copy number variation sequencing for detection of chromosomal abnormalities associated with spontaneous miscarriage. Ultrasound Obstet Gynecol.

[12] Hsu LY, Benn PA (1999). Revised guidelines for the diagnosis of mosaicism in amniocytes. Prenat Diagn.

[13] Liu J, Hu H, Ma N, Jia Z, Zhou Y, Hu J, et al. A de novo duplication of chromosome 9q34.13-qter in a fetus with Tetralogy of Fallot Syndrome. Mol Cytogenet. 2016;9:54. 10.1186/s13039-016-0267-3 PMC4960742 27462370

[14] Zhu X, Li J, Ru T, Wang Y, Xu Y, Yang Y (2016). Identification of copy number variations associated with congenital heart disease by chromosomal microarray analysis and next-generation sequencing. Prenat Diagn.

[15] Li H, Durbin R (2009). Fast and accurate short read alignment with Burrows-Wheeler transform. Bioinformatics.

[16] Eggermann T, Soellner L, Buiting K, Kotzot D (2015). Mosaicism and uniparental disomy in prenatal diagnosis. Trends Mol Med.

[17] Taylor TH, Gitlin SA, Patrick JL, Crain JL, Wilson JM, Griffin DK (2014). The origin, mechanisms, incidence and clinical consequences of chromosomal mosaicism in humans. Hum Reprod Update.

[18] Carey L, Scott F, Murphy K, Mansfield N, Barahona P, Leigh D (2014). Prenatal diagnosis of chromosomal mosaicism in over 1600 cases using array comparative genomic hybridization as a first line test. Prenat Diagn.

[19] Hsu LY, Yu MT, Richkind KE, Van Dyke DL, Crandall BF, Saxe DF (1996). Incidence and significance of chromosome mosaicism involving an autosomal structural abnormality diagnosed prenatally through amniocentesis: a collaborative study. Prenat Diagn.

[20] Lebo RV, Novak RW, Wolfe K, Michelson M, Robinson H, Mancuso MS (2015). Discordant circulating fetal DNA and subsequent cytogenetics reveal false negative, placental mosaic, and fetal mosaic cfDNA genotypes. J Transl Med.

[21] Ballif BC, Rorem EA, Sundin K, Lincicum M, Gaskin S, Coppinger J (2006). Detection of low-level mosaicism by array CGH in routine diagnostic specimens. Am J Med Genet A.

[22] Pinto IP, Minasi LB, Steckelberg R, da Silva CC, da Cruz AD. Mosaic Tetrasomy of 9p24.3q21.11 postnatally identified in an infant born with multiple congenital malformations: a case report. BMC Pediatr. 2018;18(1):298. 10.1186/s12887-018-1275-8 PMC6128999 30193577

[23] Zahir FR, Marra MA. Use of Affymetrix Arrays in the Diagnosis of Gene Copy-Number Variation. Curr Protoc Hum Genet. 2015;85:8 13 1–8 13 13. 10.1002/0471142905.hg0813s85 25827348

[24] Cohen K, Tzika A, Wood H, Berri S, Roberts P, Mason G (2015). Diagnosis of fetal submicroscopic chromosomal abnormalities in failed array CGH samples: copy number by sequencing as an alternative to microarrays for invasive fetal testing. Ultrasound Obstet Gynecol.

[25] Wang JC, Radcliff J, Coe SJ, Mahon LW (2019). Effects of platforms, size filter cutoffs, and targeted regions of cytogenomic microarray on detection of copy number variants and uniparental disomy in prenatal diagnosis: Results from 5026 pregnancies. Prenat Diagn.

[26] Nowinski GP, Van Dyke DL, Tilley BC, Jacobsen G, Babu VR, Worsham MJ (1990). The frequency of aneuploidy in cultured lymphocytes is correlated with age and gender but not with reproductive history. Am J Hum Genet.

[27] Chen CP, Chern SR, Chen YN, Wu PS, Yang CW, Chen LF (2015). Mosaic trisomy 15 at amniocentesis: Prenatal diagnosis, molecular genetic analysis and literature review. Taiwan J Obstet Gynecol.

[28] Chen CP, Hsu CY, Chern SR, Wu PS, Chen SW, Wang W (2020). Prenatal diagnosis of mosaic trisomy 8 by amniocentesis in a fetus with ventriculomegaly and dysgenesis of the corpus callosum. Taiwan J Obstet Gynecol.

[29] Chen CP, Su YN, Chern SR, Chen YT, Wu PS, Su JW (2012). Mosaic trisomy 2 at amniocentesis: prenatal diagnosis and molecular genetic analysis. Taiwan J Obstet Gynecol.

[30] Chen CP, Huang MC, Chern SR, Wu PS, Chen SW, Chuang TY (2019). Mosaic trisomy 22 at amniocentesis: Prenatal diagnosis and literature review. Taiwan J Obstet Gynecol.

