# Supplementary material for: Integrated CNV-seq, karyotyping and SNP-array analyses for effective prenatal diagnosis of chromosomal mosaicism
Source: BMC Med Genomics. 2021 Feb 25;14:56. doi: 10.1186/s12920-021-00899-x (PMC7905897; doi:10.1186/s12920-021-00899-x)
Supplement: Supplementary file 1 — Additional file 1. Table S1: Chromosome and CMA results on excluded mosaic cases of large cryptic genomic rearrangements.. [file 12920_2021_899_MOESM1_ESM.docx]

**Table S1. Chromosome and CMA results on excluded mosaic cases of large cryptic genomic rearrangements.**

| **Case No.** | **Sample type** | **Age** | **Indication** | **Chromosome (culture) Result** | **Copy number of reference chromosome** | | |
| --- | --- | --- | --- | --- | --- | --- | --- |
|  |  |  |  |  | **Karyotyping** | **CMA** | **CNV-seq** |
| 68 | AF | 22 | Failed NIPS; aUS (cerebral ventriculomegaly) | 46,X,+mar[4]/45,X[46] | / | arr[hg19] Yq11.222q11.23  (21,035,823-28,799,654)×0 arr[hg19] Yp11.2q11.21  (3,947,120-19,629,707)×2 arr[hg19] 6q16.2(99,929,316-100,502,766)×1 | N/A |
| 69 | AF | 27 | aMSS (T21) | 47,XX,+mar[5]/46,XX[88] | / | arr[hg19] 16p11.2  (32,848,887-34,420,775)×3 | N/A |
| 70 | AF | 40 | AMA, abnormal NIPS (T18) | 47,XX,+mar[26]/46,XX[24] | / | arr[hg19] 18p11.32p11.21  (136,227-15,170,636)×3 | N/A |
| 71 | AF | 32 | Abnormal NIPS (X-) | 45,X[39]/46X,i(X)(q10)[16] | / | arr[hg19] Xp22.33p11.21  (168,551- 56,143,644)× 1 | N/A |
| 72 | AF | 25 | Abnormal NIPS (X-) | 45,X[29]/46,X,i(X)(q10)[21] | / | arr[hg19] Xp22.33p11.21  (168,551-56,047,110)× 1 | N/A |

Abbreviations: CMA, chromosomal microarray analysis; AF, amniotic fluid; AMA, advance maternal age; aUS, abnormal ultrasound; T21, trisomy 21; hMSS, high-risk of maternal serum screening; NIPS, non-invasive prenatal screening; N/A, not available.
