# Supplementary material for: Integrated CNV-seq, karyotyping and SNP-array analyses for effective prenatal diagnosis of chromosomal mosaicism
Source: BMC Med Genomics. 2021 Feb 25;14:56. doi: 10.1186/s12920-021-00899-x (PMC7905897; doi:10.1186/s12920-021-00899-x)
Supplement: Supplementary file 2 — Additional file 2. Figure S1: Case 18. Panel A. CMA analysis of uncultured AF samples shows mosaic trisomy 8 (~24%). Panel B. CNV‐seq profile of the same sample shows a slightly lower ratio of mosaic trisomy 8 (~18%). The blue line represents the mean copy number and the black box represents the centromere. [file 12920_2021_899_MOESM2_ESM.pdf]

Case 18

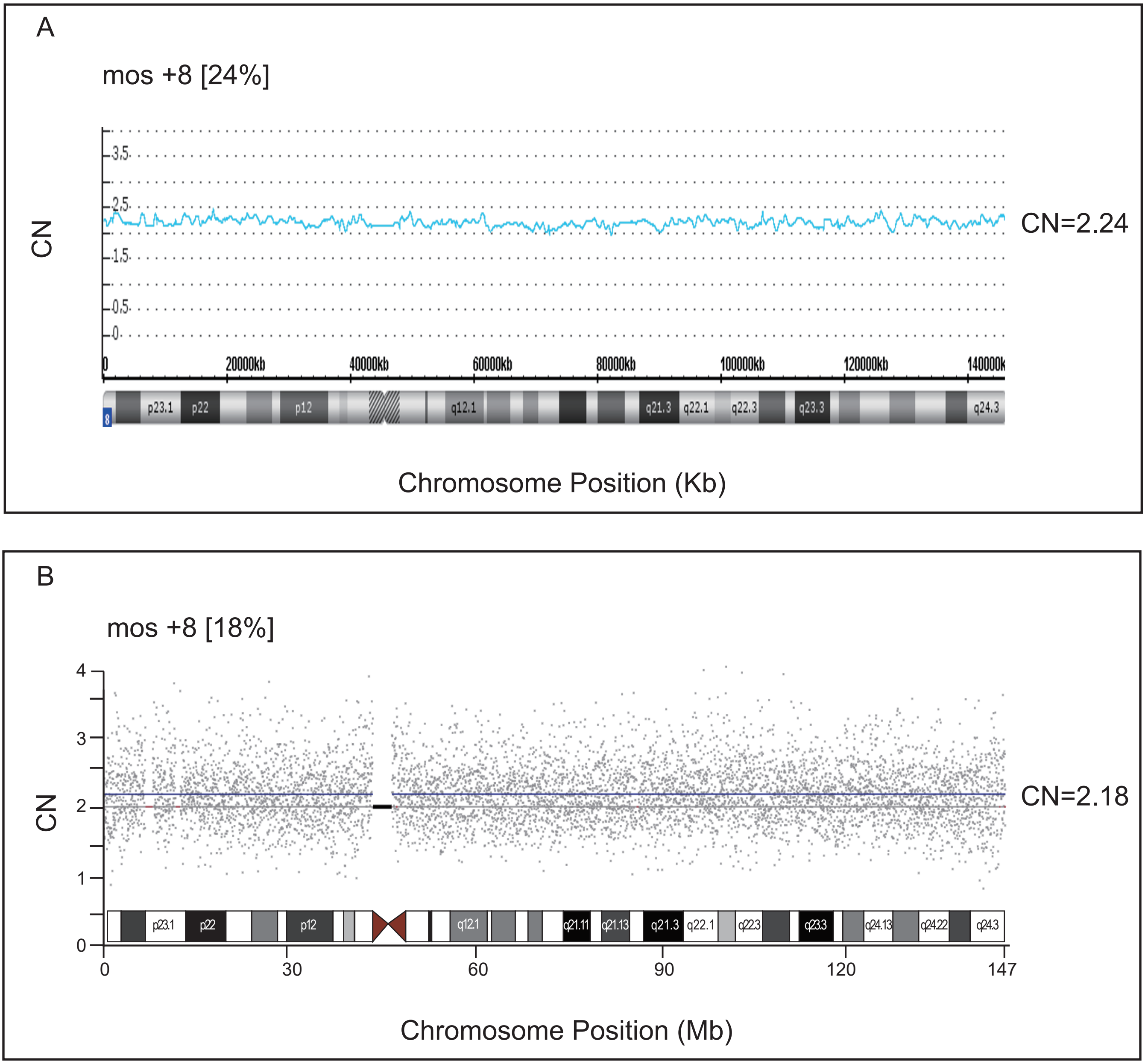


Figure S1.
Case 18.
Panel A. CMA analysis of uncultured AF samples shows mosaic trisomy 8 (~24%).
Panel B. CNV-seq profile of the same sample shows a slightly lower ratio of mosaic trisomy 8 (~18%).
The blue line represents the mean copy number and the black box represents the centromere.
