# Supplementary material for: Integrated CNV-seq, karyotyping and SNP-array analyses for effective prenatal diagnosis of chromosomal mosaicism
Source: BMC Med Genomics. 2021 Feb 25;14:56. doi: 10.1186/s12920-021-00899-x (PMC7905897; doi:10.1186/s12920-021-00899-x)
Supplement: Supplementary file 3 — Additional file 3. Figure S2: Cases 36 and 41. Panel A. The CMA analysis of uncultured AF samples shows a normal result. The CNV‐seq profile of the same AF sample shows a low ratio of mosaic monosomy X (~8%). The CNV‐seq profiles of the maternal and fetal center of placenta show monosomy X with the level of 11% and 83% mosaicism. The blue line represents the mean copy number and the black box represents the centromere. Panel B. The CMA analysis on uncultured AF samples shows a normal result. The CNV‐seq profiles of the same AF samples shows a low ratio of mosaic monosomy X (~8%). The CNV‐seq profiles of the maternal and fetal center of placenta show monosomy X with the level of 15% and 78% mosaicism. The blue line represents the mean copy number and the black box represents the centromere. [file 12920_2021_899_MOESM3_ESM.pdf]

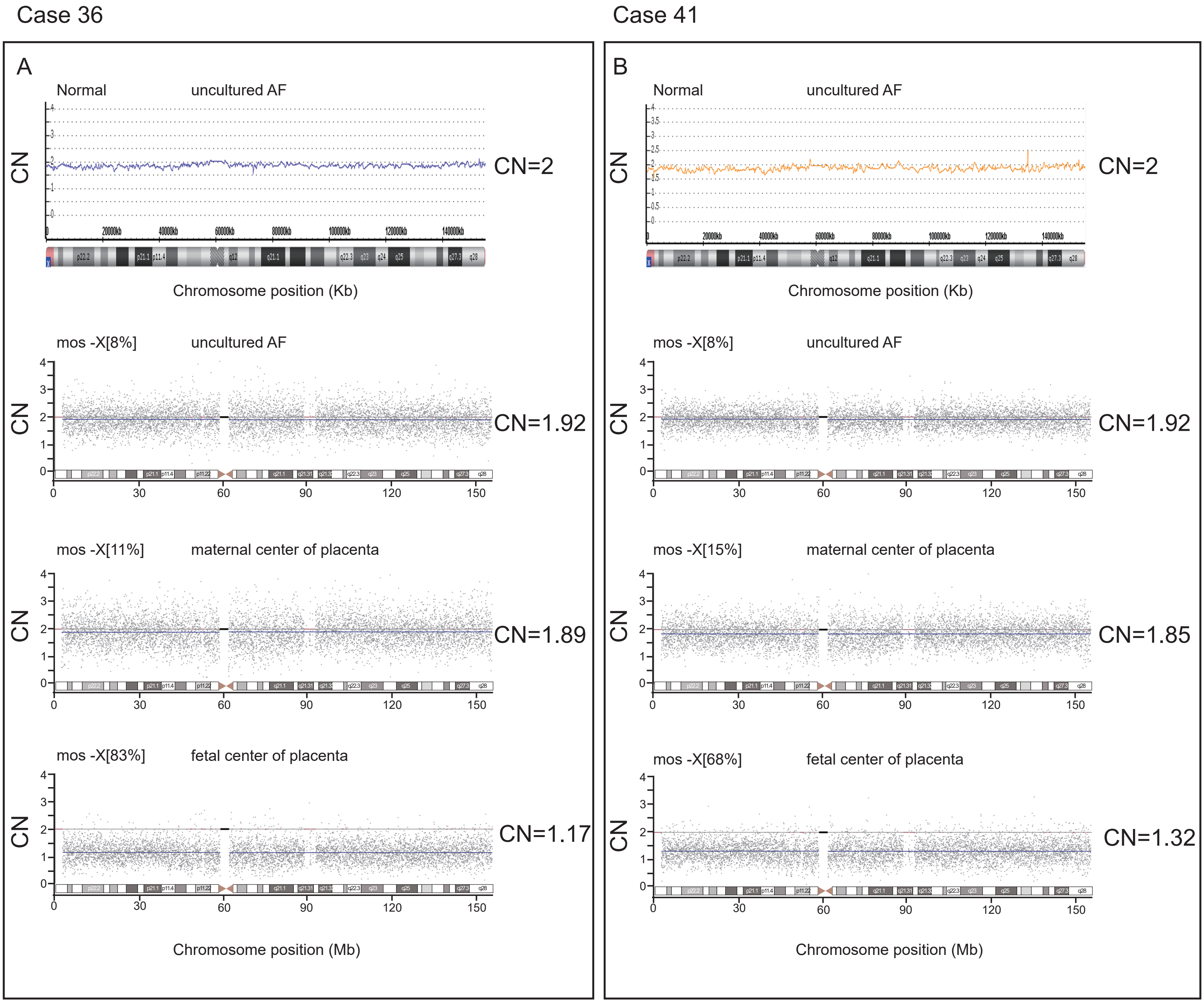

Figure S2.
Cases 36 and 41.
Panel A. The CMA analysis of uncultured AF samples shows a normal result. T
he CNV-seq profile of the same AF sample shows a low ratio of mosaic monosomy X (~8%).
The CNV-seq profiles of the maternal and fetal center of placenta show monosomy X with the level of 11% and 83% mosaicism.

Panel B. The CMA analysis on uncultured AF samples shows a normal result. The CNV-seq profiles of the same AF samples shows a low ratio of mosaic monosomy X (~8%). The CNV-seq profiles of the maternal and fetal center of placenta show monosomy X with the level of 15% and 78% mosaicism.
The blue line represents the mean copy number and the black box represents the centromere.
