# Supplementary material for: Integrated CNV-seq, karyotyping and SNP-array analyses for effective prenatal diagnosis of chromosomal mosaicism
Source: BMC Med Genomics. 2021 Feb 25;14:56. doi: 10.1186/s12920-021-00899-x (PMC7905897; doi:10.1186/s12920-021-00899-x)
Supplement: Supplementary file 4 — Additional file 4. Figure S3. Case 63 (normal karyotype). CMA and CNV-seq results were discordant with karyotyping. Panel A. CMA analysis on uncultured cord blood sample shows a 20.44 Mb mosaic duplication at chromosome 8p21.3p11.21 (~ 23% of cells) marked by arrow. Panel B. CNV-seq shows a mosaic 8p22p11.1 duplication of 25.3 Mb (~23% of cells). Positions of CNVs are indicated by the dashed boxes. [file 12920_2021_899_MOESM4_ESM.pdf]

Case 63

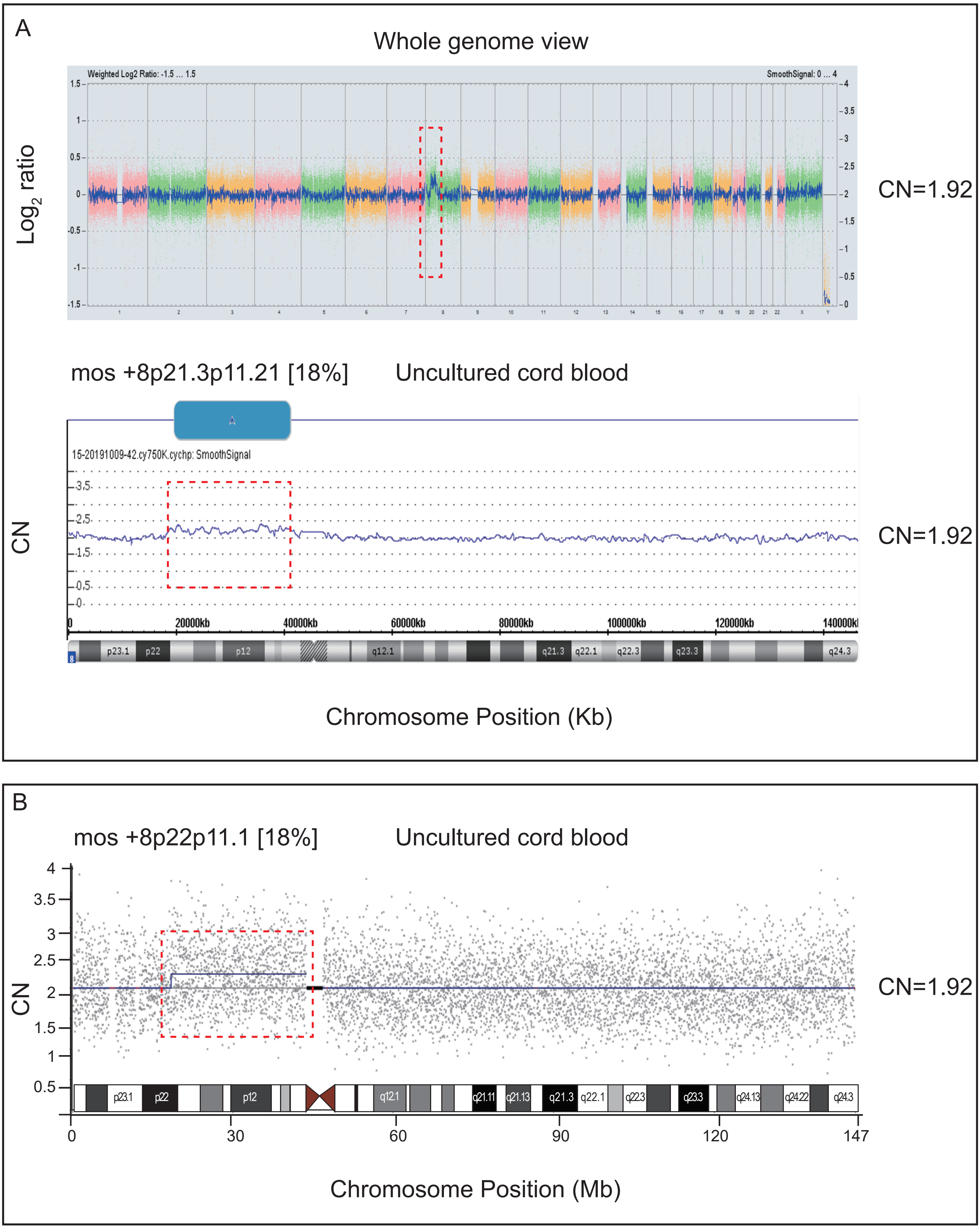

Figure S3.
Case 63 (normal karyotype). CMA and CNV-seq results were discordant with karyotyping.
Panel A. CMA analysis on uncultured cord blood sample shows a 20.44 Mb mosaic duplication at chromosome 8p21.3p11.21 (~ 23% of cells)
Panel B. CNV-seq shows a mosaic 8p22p11.1 duplication of 25.3 Mb (~23% of cells).
Positions of CNVs are indicated by the dashed boxes.
