# Supplementary material for: Integrated CNV-seq, karyotyping and SNP-array analyses for effective prenatal diagnosis of chromosomal mosaicism
Source: BMC Med Genomics. 2021 Feb 25;14:56. doi: 10.1186/s12920-021-00899-x (PMC7905897; doi:10.1186/s12920-021-00899-x)
Supplement: Supplementary file 5 — Additional file 5. Figure S4: Case 64. Chromosomal mosaicism for Xp2.33q21.31 detected by CMA and CNV-Seq. Panel A. CMA result. Panel B. CNV-Seq result. Blue lines on sequencing plots represent mean copy number changes. Panel C. Karyotype showing Xp deletion. [file 12920_2021_899_MOESM5_ESM.pdf]

Case 64

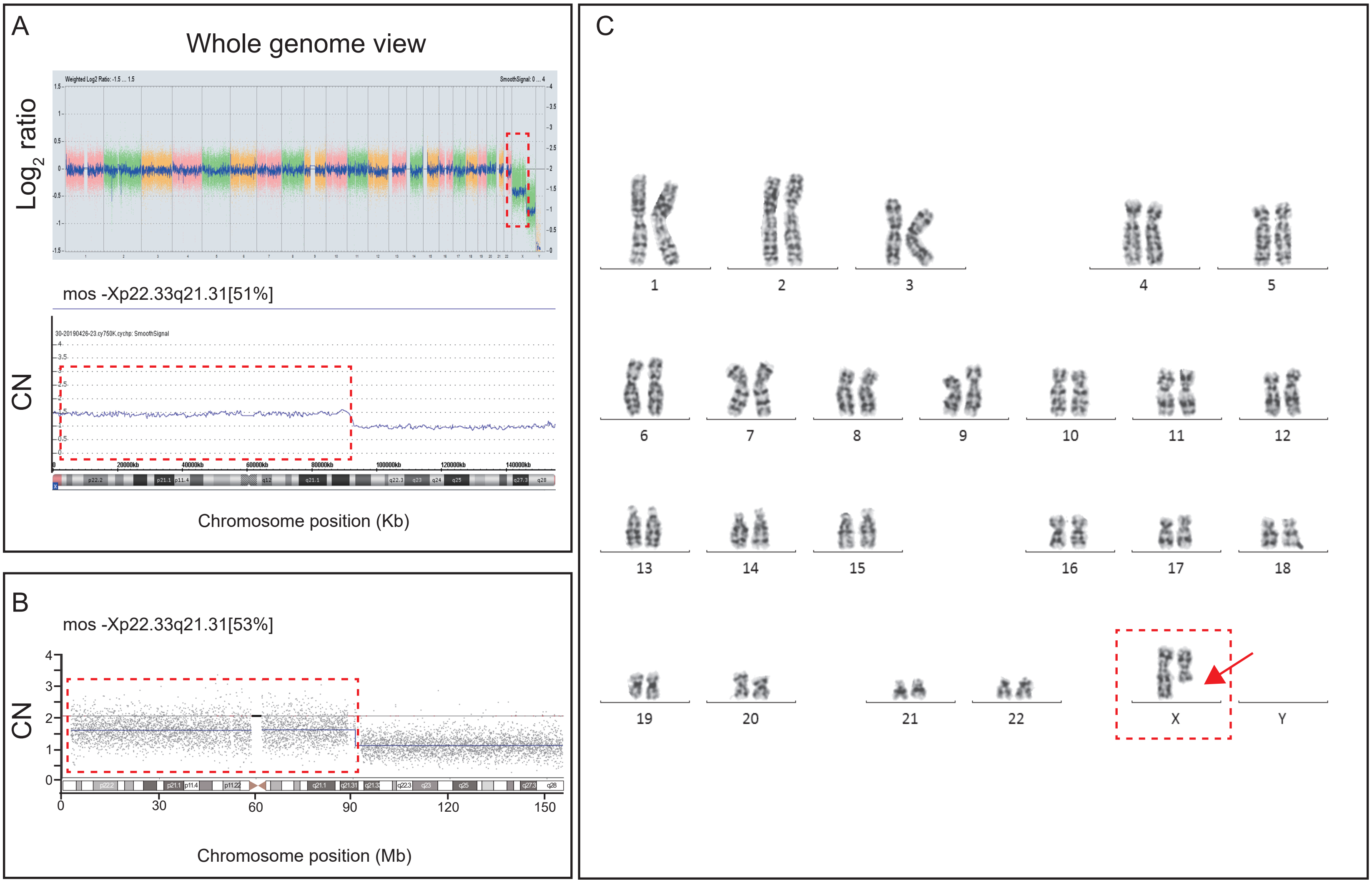

Figure S4.

Case 64. Chromosomal mosaicism for Xp2.33q21.31 detected by CMA and CNV-Seq.

Panel A. CMA result. Panel

Panel B. CNV-Seq result. Blue lines on sequencing plots represent mean copy number changes.

Panel C. Karyotype showing Xp deletion.

Positions of CNVs are indicated by the dashed boxes.
