# Supplementary material for: Integrated CNV-seq, karyotyping and SNP-array analyses for effective prenatal diagnosis of chromosomal mosaicism
Source: BMC Med Genomics. 2021 Feb 25;14:56. doi: 10.1186/s12920-021-00899-x (PMC7905897; doi:10.1186/s12920-021-00899-x)
Supplement: Supplementary file 6 — Additional file 6. Figure S5: Case 65. Mosaic duplication of Yq11.222q11.223 detected by CMA and CNV-Seq. Panel A. CMA result. Panel B. CNV-Seq result. Blue lines on sequencing plots represent mean copy number changes. Panel C. Karyotype showing Yq duplication. Positions of CNVs are indicated by the dashed boxes. [file 12920_2021_899_MOESM6_ESM.pdf]

Case 65

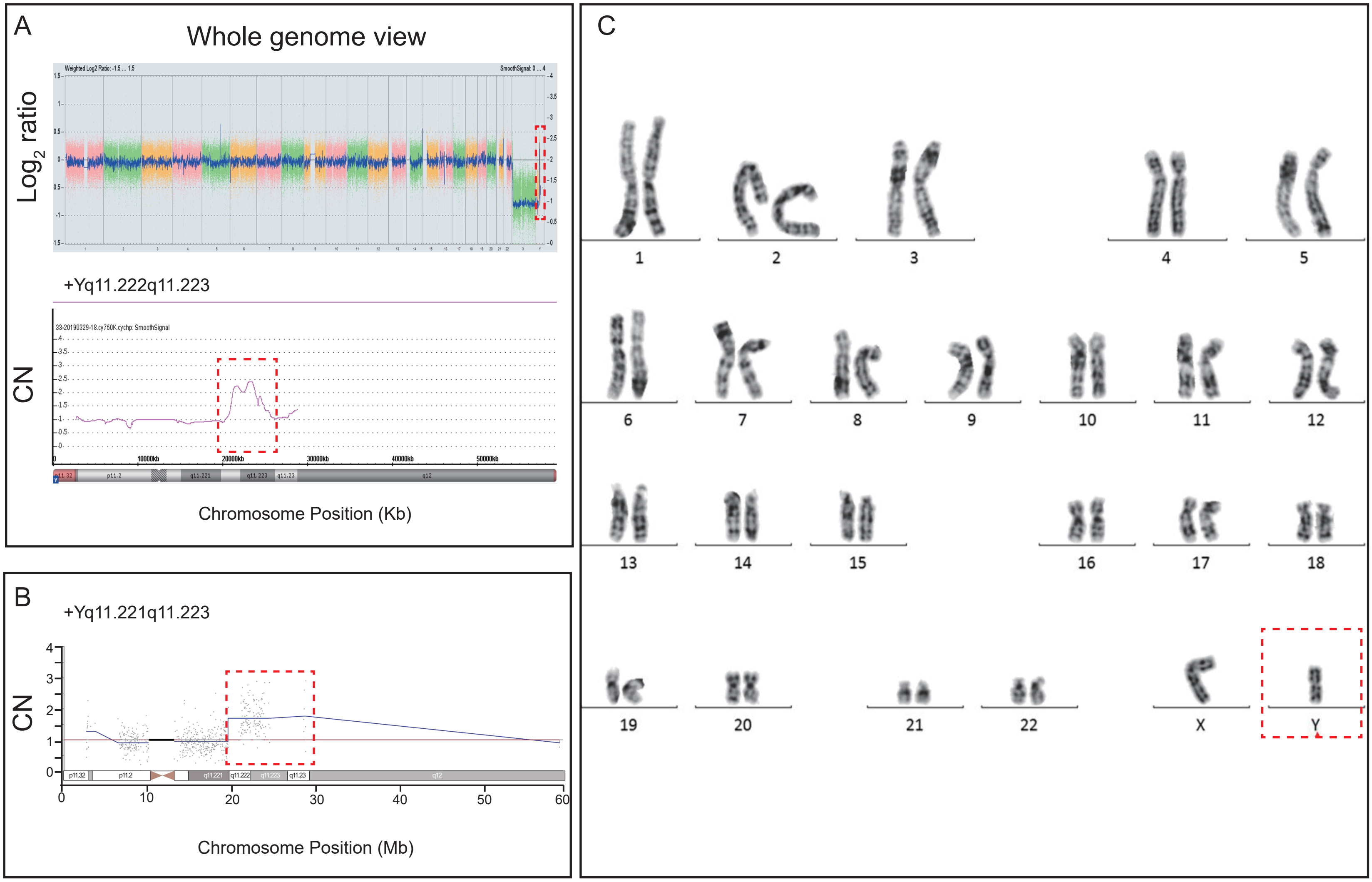

Figure S5.
Case 65. Mosaic duplication of Yq11.222q11.223 detected by CMA and CNV-Seq.
Panel A. CMA result.
Panel B. CNV-Seq result. Blue lines on sequencing plots represent mean copy number changes.
Panel C. Karyotype showing Yq duplication.
Positions of CNVs are indicated by the dashed boxes.
