# Supplementary material for: Integrated CNV-seq, karyotyping and SNP-array analyses for effective prenatal diagnosis of chromosomal mosaicism
Source: BMC Med Genomics. 2021 Feb 25;14:56. doi: 10.1186/s12920-021-00899-x (PMC7905897; doi:10.1186/s12920-021-00899-x)
Supplement: Supplementary file 8 — Additional file 8. Figure S7: Case 67. Mosaic trisomy 21 and mosaic duplication of Xp11.21q28 detected CMA and CNV-Seq. Panel A. CMA result. Panel B. CNV-Seq result. Blue lines on sequencing plots represent mean copy number changes. Panel C. Karyotype showing trisomy 21 and XXX. Positions of CNVs are indicated by the dashed boxes. [file 12920_2021_899_MOESM8_ESM.pdf]

Case 67

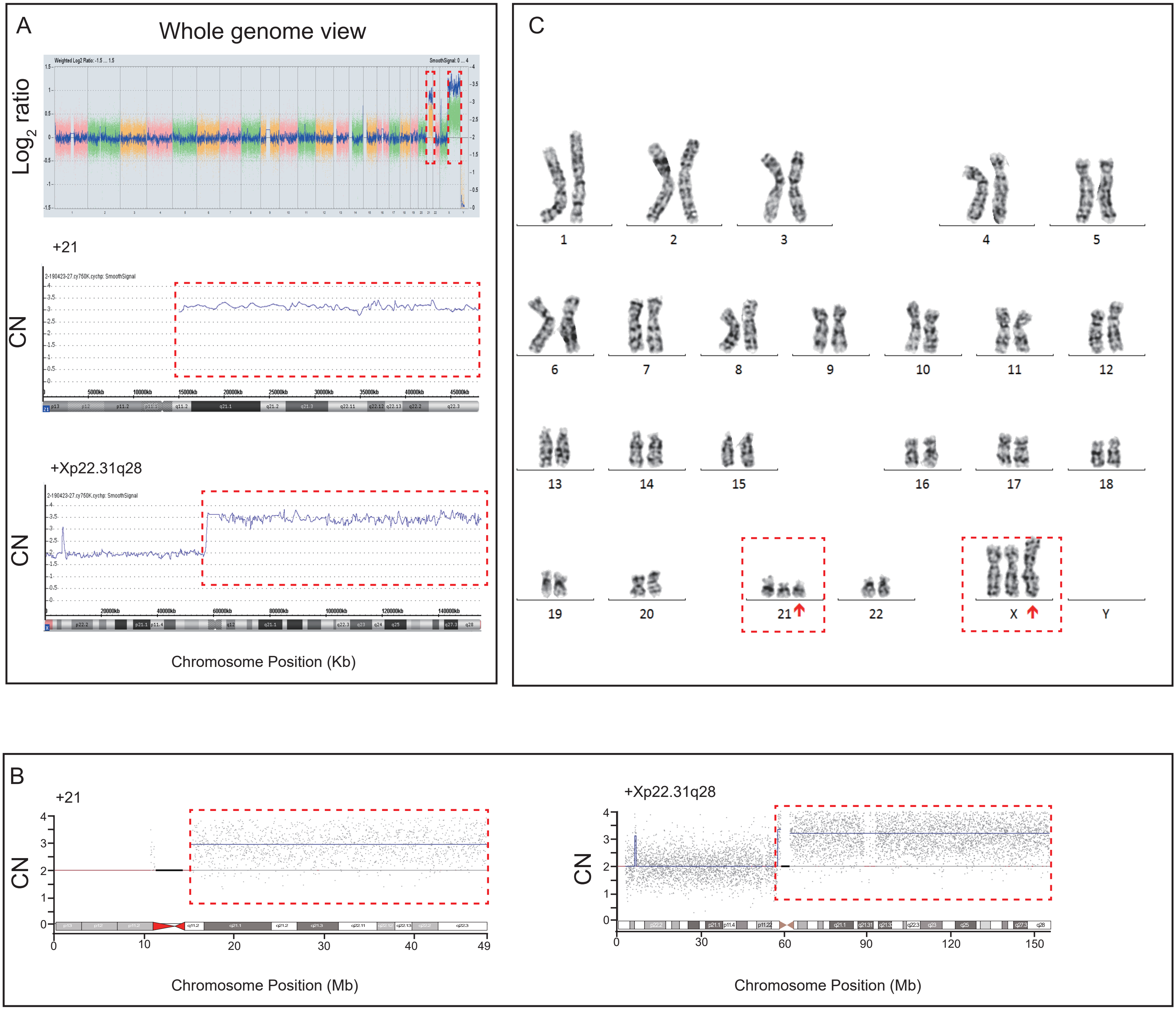

Figure S7.
Case 67. Mosaic trisomy 21 and mosaic duplication of Xp11.21q28 detected CMA and CNV-Seq.
Panel A. CMA result.
Panel B. CNV-Seq result. Blue lines on sequencing plots represent mean copy number changes.
Panel C. Karyotype showing trisomy 21 and XXX.
Positions of CNVs are indicated by the dashed boxes.
